# Physician-Friendly Machine Learning: A Case Study with Cardiovascular Disease Risk Prediction

**DOI:** 10.3390/jcm8071050

**Published:** 2019-07-18

**Authors:** Meghana Padmanabhan, Pengyu Yuan, Govind Chada, Hien Van Nguyen

**Affiliations:** Department of Electrical and Computer Engineering, University of Houston, Houston, TX 77004, USA

**Keywords:** artificial intelligence, clinical domain, auto machine learning, cardiovascular disease prediction, physician-friendly machine learning

## Abstract

Machine learning is often perceived as a sophisticated technology accessible only by highly trained experts. This prevents many physicians and biologists from using this tool in their research. The goal of this paper is to eliminate this out-dated perception. We argue that the recent development of auto machine learning techniques enables biomedical researchers to quickly build competitive machine learning classifiers without requiring in-depth knowledge about the underlying algorithms. We study the case of predicting the risk of cardiovascular diseases. To support our claim, we compare auto machine learning techniques against a graduate student using several important metrics, including the total amounts of time required for building machine learning models and the final classification accuracies on unseen test datasets. In particular, the graduate student manually builds multiple machine learning classifiers and tunes their parameters for one month using scikit-learn library, which is a popular machine learning library to obtain ones that perform best on two given, publicly available datasets. We run an auto machine learning library called auto-sklearn on the same datasets. Our experiments find that automatic machine learning takes 1 h to produce classifiers that perform better than the ones built by the graduate student in one month. More importantly, building this classifier only requires a few lines of standard code. Our findings are expected to change the way physicians see machine learning and encourage wide adoption of Artificial Intelligence (AI) techniques in clinical domains.

## 1. Introduction

Machine learning and artificial intelligence (AI) have witnessed tremendous progress in the past five years. AI algorithms have gained significant interest from clinical researchers. As an example, a recent survey indicates that nearly half of the interviewed healthcare organizations are using or planning to use artificial intelligence in imaging [1]. Unfortunately, developing machine learning algorithms traditionally requires a significant amount of time and understanding of how the underlying algorithms work. For example, tuning and training of deep neural networks take weeks to months. Most state-of-the-art deep networks have been manually designed by human experts who have advanced degrees and long-term training in computer science and artificial intelligence [2,3,4,5]. Such requirements pose a great challenge for clinical researchers who want to use AI tools to validate important biomedical questions.

Motivated by this limitation, researchers recently investigated more automated AI techniques [6,7,8,9,10,11,12]. These methods are collectively known as Auto Machine Learning (AutoML). The idea is to automate the process of building an AI model that gives competitive performances on any given dataset. This includes automation of data pre-processing, feature extraction, hyper-parameter tuning, and algorithm selection. Here, a feature means a compact vector containing information about the input data essential for making the final prediction. The emergence of AutoML is potentially transformative to the biomedical and clinical domains. By removing the high technical barrier, AutoML could enable physicians to use AI techniques more broadly in their work and research.

AutoML can be viewed as the end-to-end process of searching for the best AI model configuration on an arbitrarily given dataset. Each configuration is the result of making multiple choices regarding which algorithm, optimization method, or hyper-parameter to use. Due to the vast number of configurations in the search space, finding the best model is computationally expensive. As computer processing power improves thanks to advanced technologies such as graphics processing units (GPU) and tensor processing units (TPU), and more efficient search algorithms, AutoML methods have been able to scale up dramatically. Recent papers showed that classifiers built by automated techniques have reached or even surpassed those designed by human experts. Notable examples include AmoebaNet [13] which outperforms most state-of-the-art architectures on a large-scale natural image dataset [14], and Auto-Sklearn [12] which has shown competitive performances on multiple heterogeneous datasets. Major technological companies, including Google and Microsoft, aware of the vast potential of AutoML across industries, have recently started to build their own AutoML platforms. Despite the great potential, AutoML has not been well-studied in biomedical applications. Our paper will make the following contributions:

- For the first time, we investigate the use of AutoML for building classifiers of cardiovascular diseases.
- We compare AutoML performances against that of a graduate student with significant experience in machine learning and computer programming.
- We provide extensive experimental results on two cardiovascular datasets.

This study will inform physicians and biomedical researchers on an important and emerging machine learning tool. Our findings will shed light on what benefit AutoML can bring, how easy it is to use the tool, and how well it performs compared to a human expert. Although we evaluate our method on cardiovascular data, the findings are expected to hold for other kinds of biomedical data.

Given that cardiovascular diseases are the leading cause of deaths in the world today [15] and the availability of tremendous amount of cardiovascular data, there have been numerous studies in the past to get machine learning models to deduce patterns in the data to allow for early detection of heart diseases. Multiple standalone machine learning models and hybrid models have been proposed [16].

Vembandasamy et al. [17] propose the use of Naive Bayes classifier for prediction of heart disease on a dataset from a leading diabetic research institute in Chennai, India containing 500 records and 10 attributes. The Naive Bayes classifier attained accuracy of 86.4%. Shouman et al. [18] propose the Decision Tree classifier on the benchmark Heart UCI (University of California, Irvine, CA, USA) dataset by applying several tuning techniques to Decision Trees like different combinations of discretization, tree types, voting, etc. to identify a reliable, robust and accurate method of classification. The final reported accuracy is 84.1%. Srinivas et al. [19] propose more complicated data mining algorithms. The technique involves the extraction of significant patterns from the dataset, choosing patterns with values greater than a prescribed threshold and using five different mining goals. The reported accuracy is 83.7%. Tomar et al. [20] use Least Squares Twin Support Vector Machines [21] for diagnosis of heart diseases using the grid-search approach for hyperparameter selection and F-scores as the evaluation metric on the heart UCI dataset. Reported accuracy is 85.59%. Several ensemble classifiers, which are a weighted combination of simple classifiers have also been seen to work well with heart disease prediction. Pouriyeh et al. [22] use the Decision Tree classifier, Naïve Bayes classifier, Multilayer Perceptron, K-Nearest Neighbor classifier, Single Conjunctive Rule Learner and Radial Basis Function with Support Vector Machines both individually and in combination on the Heart UCI dataset. In addition, bagging, boosting and stacking techniques have been applied on each of the above-mentioned classifiers. The best performing classifier was reported to be a combination of the Support Vector Machine and the Multilayer Perceptron and the reported accuracy is 84.81%. Bashir et al. [23] propose the use of an ensemble classifier that uses an enhanced bagging approach with the multi-objective weighted voting scheme. Five different base classifiers including Naïve Bayes, linear regression, quadratic discriminant analysis, instance-based learner and support vector machines are used. Five different heart disease datasets are used. The experimental evaluation shows that the proposed framework achieves diagnosis accuracy of 84.16%.

There are several challenges associated with manually training and evaluating machine learning models. Most important is the difficulty in correctly identifying the nature (continuous or categorical) of all features to preprocess them accordingly before passing them into machine learning models. The required expertise and time associated with this task are also significantly high. This study proposes to use auto machine learning as a solution to the above-mentioned problems and attempts to quantify the performance and time benefits that auto machine learning has to offer over a manually built solution.

Section 2 of this paper discusses the processes and techniques involved in the experimental stage. Section 2.1 describes Auto-Sklearn and the techniques adopted by it to find and fine-tune the machine learning model best suited for the dataset. Section 2.2 provides a description of the datasets, the nature of preprocessing and the train-test procedure applied on each of them by the graduate student. Furthermore, Section 2.3 goes on to elucidate challenges that the graduate student faced during manual training and how they were addressed. Section 3 discusses the results drawn from manual training and Auto-Sklearn and provides a comparison between the corresponding evaluation metrics obtained post training. Finally, Section 4, the concluding section, describes how the ease of use and superior performance of the AutoML tool as described in this paper could greatly impact the clinical domain.

## 2. Materials and Methods

Our experiments focus on evaluating the performances of AutoML techniques on two cardiovascular datasets. In particular, we will use Auto-Sklearn [12], which is one of the state-of-the-art generic AutoML frameworks. Auto-Sklearn has consistently won several AutoML competitions over the years. Moreover, its user interface is friendly and a non-technical person without much prior knowledge in machine learning can quickly achieve mastery. In what follows, we will provide a brief description of Auto-Sklearn and the experimental setup.

### 2.1. Algorithmic Description of Auto-Sklearn

**Figure 1:**
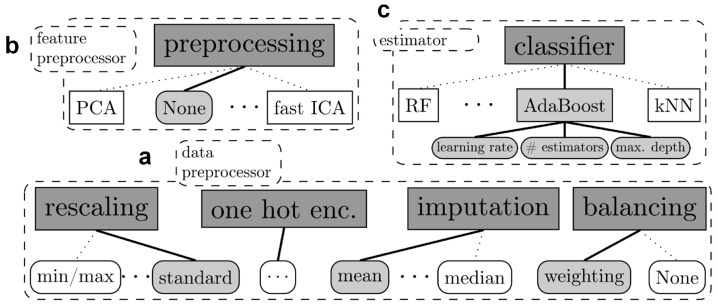
The Auto-Sklearn pipeline [12] contains three main building blocks: (**a**) Data preprocessor, (**b**) Feature preprocessor, and (**c**) Estimator or machine learning algorithms.

Auto-Sklearn was proposed in [12]. The name was motivated by Scikit-Learn [24], a popular generic machine learning toolbox. Auto-Sklearn automates the process of building an AI model by utilizing a large number of machine learning classifiers (14 in total) and pre-processing steps (14 feature processing methods, and four data preprocessing methods) in the Scikit-Learn toolbox. This includes logistic regressions, support vector machines, random forests [25], boosting, and neural networks. Figure 1 shows the graphical illustration of the pipeline. Given the training data, Auto-Sklearn first selects an appropriate set of data preprocessing steps such as rescaling or imputation of missing values. It then passes the processed data to the feature processing block, which further normalizes the data or reduces their dimensions using standard techniques such as principal component analysis [26] and independent component analysis [27]. Finally, data are passed to the estimator block, which selects and trains machine learning algorithms to predict desirable outputs from input data samples.

Auto-Sklearn defines AutoML as the process of automatically producing test-set predictions (without any human intervention) given a fixed computational budget. Here, computational budget means computer run time or computer memory usage. Auto-Sklearn combines traditional machine learning techniques with a Bayesian optimization framework to search for the best combination of AI models and parameters. It also introduces several notable improvements compared to previous approaches [28]. First, it uses prior experience on other datasets to create a good model initialization for a new dataset. The central intuition is that domain experts derive knowledge from previous tasks. Motivated by this observation, Auto-Sklearn employs a similar strategy. It collects a set of 38 *meta-features*, or vector descriptions of dataset properties that would help to determine appropriate algorithms that would likely perform well on a particular dataset. Examples of meta-features include statistics about the number of data samples, data dimensions, classes, and skewness. Based on these features, Auto-Sklearn makes a rough suggestion for what algorithms, pre-processing, and other hyper-parameters will work well on a particular dataset. Bayesian optimization further refines and improves the model. Second, instead of outputting one model, Auto-Sklearn uses a weighted combination of multiple best-performing models. This is similar to the ensemble method in random forests [25] that combines multiple random trees to reduce the prediction variance. Empirical studies found that this modification significantly improves the robustness of the final model [12].

**Figure 2:**
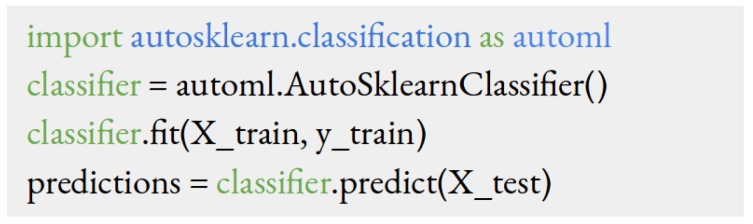
Python code for using Auto-Sklearn to train a classifier for any dataset.

A non-technical person will find Auto-Sklearn intuitive and easy to learn. Figure 2 shows the code for training a classifier for an arbitrary dataset. It essentially contains only four lines of code. The first line loads the Auto-Sklearn library, assuming that this library is already pre-installed in the computer. The second line of code creates an instance of the classifier. One can think of this as a placeholder for the final classifier. The third line of code calls the function **.fit** to train (also known as fitting) the final classifier given the training data **X_train** and the corresponding labels **y_train**. The last line calls the function **.predict** to make the predictions on the test data **X_test**.

### 2.2. Process of Building AutoML Models

The process of building an AutoML model contains two main steps. The first step is to prepare data into an appropriate format and load them into the computer’s memory. In our case, we store our training and test data in **.csv** tables whose rows and columns represent different patients and features, respectively. We then load those tables into the computer memory using the standard **csv** library in Python. Readers can refer to [29] for more information about the process of reading **.csv** files. Let **X_train** and **y_train** respectively denote the training features and labels that we have loaded into the computer memory. The second step is to run AutoML code described in Figure 2. This script will import Auto-Sklearn and build a classifier to predict the outcome for each patient. The classifier can be used to make prediction on new patients through the command **classifier.predict(x_test)**, where **x_test** is the test data from a new patient as shown in Figure 2. In this paper, we use a computer with a 16-core processor (i9-7960X, 16 Cores, 2.80 GHz) and a Titan-V graphic processing unit (NVIDIA GeForce Titan V, 12 GB HBM2 Memory, NVIDIA, Santa Clara, CA, USA). However, we believe that any modern computers will not have problems with running the AutoML code since Auto-Sklearn is written in Python which can compile across different hardware platforms and operating systems.

### 2.3. Datasets and Manual Preprocessing by a Graduate Student

Training, testing and evaluation are performed on two different cardiovascular datasets, the Heart UCI (University of California, Irvine, CA, USA) dataset and the Cardiovascular Disease Dataset. In what follows, the two datasets, including the nature and meaning of each of their features, are described. Given the vast difference in the nature of these two datasets, subsequent sections address the training procedures and the challenges independently for the two datasets.

#### 2.3.1. Dataset Description

The Heart UCI dataset contains data of patient records with the target field referring to the presence or absence of heart disease. The database has 76 attributes, but only 13 attributes are used for our experiments to make our results comparable to previous machine learning papers. Table 1 shows the selected attributes and their properties. This dataset has in total 303 records, which is relatively small given that a typical machine learning dataset contains several thousand to hundreds of thousands of data points. There have been multiple works [18,30,31,32,33,34,35,36] investigating the performances of different machine learning algorithms on this dataset. The popularity of this dataset makes it easy to know how competitive the results of the graduate student are as well as how the performance of the AutoML method compares to human–experts’ systems. The target variable in this dataset is ‘Target’ in Table 1. Of the 303 records, 138 records are that of patients with Target 0 and 165 records with Target 1.

The cardiovascular disease dataset consists of 70,000 records of patients’ data with the target (Cardio) describing the presence or absence of heart disease using 11 features as described in Table 2 [37]. The input features are of three types: objective (containing factual information), examination (containing the results of a medical examination) and subjective (containing information given by the patient). The target variable in this dataset is ‘Cardio’ in Table 2. Of the 70,000 records, 35,021 records are that of patients with Cardio 0 and 34,979 records are that of patients with Cardio 1.

#### 2.3.2. Manual Data Preprocessing

Data preprocessing is important to ensure the quality of the final machine learning model. The graduate student performs a number of preprocessing steps. The steps include:

1. Looking for missing data and performing missing data imputation (both datasets did not have any missing data).
2. Identifying continuous and categorical features.
3. Identifying ordinal categorical features (where the categories have a natural ordered relationship between each other) and integer encoding them.
4. Identifying nominal categorical features (where there is no natural ordered relationship between categories), and one-hot encoding them.
5. Feature scaling, so as to bring all feature values to a similar dynamic range, which allows for faster convergence of learning algorithms optimized with the gradient descent method.
6. Feature selection, which is the process of choosing relevant features from the given set and eliminating features that do not contribute much to the prediction of the target variable. This reduces training time and improves performance. Statistical tests allow ranking features according to their relation with the target. The F-statistical test (to capture linear relationships between features and target) and mutual information test (to capture linear and nonlinear relationships between features and target) are used independently on the datasets to evaluate the best set of features. In addition, recursive feature elimination, which is a greedy optimization algorithm that intends to find the best subset of features by repeatedly creating models and ranking the model’s performance with each subset, is also used. The above-mentioned statistical techniques are applied to capture complicated and nonlinear relationships of each feature with the target. Once the best set of features are extracted from training data independently using the above techniques, models are fitted on the new feature subsets and the corresponding cross validation performances are evaluated independently and compared so as to make a fool-proof decision on which feature-selection technique works well with an algorithm.

#### 2.3.3. Train and Test Procedure

Our experiments split each dataset into three distinct sets: (1) training set, (2) validation set, and (3) test set. The graduate student uses the validation set for determining the best hyper-parameters and machine learning algorithms. Once the best model and its hyper-parameters are found for a particular dataset, the student merges the training and the validation sets together and performs training on the merged set to get the final model. Note that Auto-Sklearn does not need the validation set since the hyper-parameters are selected automatically by the framework. Therefore, we merge the training set and validation set together and use that as the training data for Auto-Sklearn. We compare the graduate student’s results to that of Auto-Sklearn using the test set, which has never been used in any way during the training step.

The Heart-UCI dataset, with only 303 samples (a sample is a data point or record in the dataset that is, one trial subject’s information), demands a careful split ratio to ensure that there are enough test samples to provide a fair representation of the dataset when the model is put to test, while at the same time having enough data samples to train and validate the model performance. One-hundred samples are set aside for testing while retaining 203 for training and validation. Cross validation is performed using the k-fold cross validation technique, wherein the data are split into k groups, trained and fitted on k-1 groups and validated on one group. This procedure repeats until all k groups are validated once. The mean performance of the model on the k folds serves as the estimated cross validation performance. k is chosen to be 5 in order to ensure that each fold is large enough to be representative of the whole dataset.

The Cardiovascular disease dataset has 70,000 records, of which 14,000 records serve as the test dataset and the remaining 56,000 records are used for training and cross validation. With most machine learning models used to train this dataset, k-fold cross validation is used with k = 10, but, in the case of certain models (specifically Neural networks, Support vector machines and Bagged K-Nearest Neighbors), k-fold cross validation consumes high execution times (due to large dataset size), and the hold out cross validation scheme (where a portion of the training data are sampled before training and serves as the cross validation set) is applied in those cases. Both datasets are randomly sampled to ensure that each target subgroup receives proper representation within sets.

The graduate student uses ten main machine learning models in Scikit-Learn to build classifiers for the two datasets. These models include the logistic regression model, the support vector machines with different kernels, the decision tree and ensemble tree models, and the boosting and bagging classifiers with appropriate models as base classifiers. Finally, an ensemble classifier that combines the best performing base classifiers is trained. The datasets are manually trained and tested over a span of 30 days until satisfactory performance that works well in terms of bias and variance is obtained. The best model found fit for the Heart UCI dataset after tuning and cross validating over 17 days is found to be the hyperparameter tuned Linear Support Vector classifier (with features selected using the Recursive Feature Selection technique), while the best model for the Cardiovascular Disease Dataset after 15 days of training was found to be the bagged and hyper-parameter tuned decision tree model.

### 2.4. Challenges during Training Faced by a Graduate Student

Heart UCI Dataset: Given the small size of the dataset, over-fitting of models to training data poses a major challenge. High variance in Logistic Regression and Support vector models is addressed by carefully tuning the regularization parameter and setting it to the value at which cross-validation performance begins to fall and training performance begins to increase rapidly.

The decision tree model also over-fits the data as expected. While careful hyper-parameter tuning of tree depth significantly improves performance, other model averaging techniques are also used to enhance generalization. Multiple ensemble tree models, which construct multiple (hyper-parameter selected) trees with sample and feature subsets, are constructed and suitably hyper-parameter tuned.

Given the many parameters (number of hidden layers, number of nodes in each of these hidden layers and the maximum number of iterations, among many others) that need to be tuned in a Multi-Layer Perceptron (or Artificial Neural Network), the challenge is tackled by manually comparing the training and validation performances of the model. The model is kept simple and small, given the high chance of over-fitting. Once the model performance is satisfactory with default maximum iterations, the model shape and size are fixed, and the maximum number of iterations is tuned to ensure the convergence of Gradient Descent.

Cardiovascular Disease Dataset: The biggest challenge associated with training this dataset is that of high training time, limiting the number of models that can be trained. Of all the trained models, the Support Vector Machine models are seen to consume the most time given their computational complexity. As a solution, Principal Component Analysis (PCA) is applied to the input features to obtain dimensionality reduction of the input and lower the computational complexity and speed up the training process.

Given the high computation time, building and evaluating models over large hyper-parameter ranges are time expensive. For example, hyper-parameter tuning of the Adaboost classifier with decision trees as the base classifier takes close to a day. Some common practices are resorted to while selecting hyper-parameters of time expensive models. For example, the value of k is set to be odd and equal to the square root of the number of samples for K-Nearest Neighbors, and the number of nodes in the hidden layer of Multi-Layer Perceptron is set to the average of input and output nodes.

### 2.5. Comparison of AutoML and Human

Qualification of the graduate student: We design several experiments to compare the performance between AutoML and the graduate student. The student is highly qualified for developing a machine learning model. First, the student has taken a machine learning course and received an A letter grade for the overall performance. The course covers in-depth machine learning theories as well as multiple programming assignments. For this reason, we expect the graduate student to serve as a strong baseline for comparison with AutoML performances. Furthermore, the machine learning models developed by this student for the two datasets produce competitive results compared to prior work as described in more detail in the next section.

Metrics for comparing AutoML and the graduate student: The student was given roughly two weeks for working on each dataset. The first dataset takes 18 days, which is three days longer than the second dataset due to additional time for setting up the project and becoming familiar with the Scikit-Learn. We study how the best results obtained by the student compare to that produced by AutoML. To gain an insight into the student’s progress, we record the classification accuracies, the areas under ROC and PR curves, and major challenges faced by the student over different days. Analyzing where the student spent most of the time and technical challenges will enable a deep understanding of how AutoML will benefit the development of machine learning models.

## 3. Results and Discussion

### 3.1. AutoML Benefits Complex Datasets More

**Figure 3:**
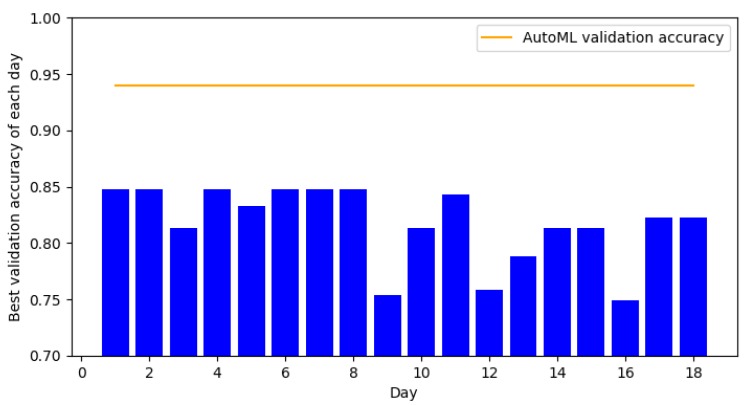
Validation accuracy over 18 days by the graduate student on the Heart UCI dataset.

**Figure 4:**
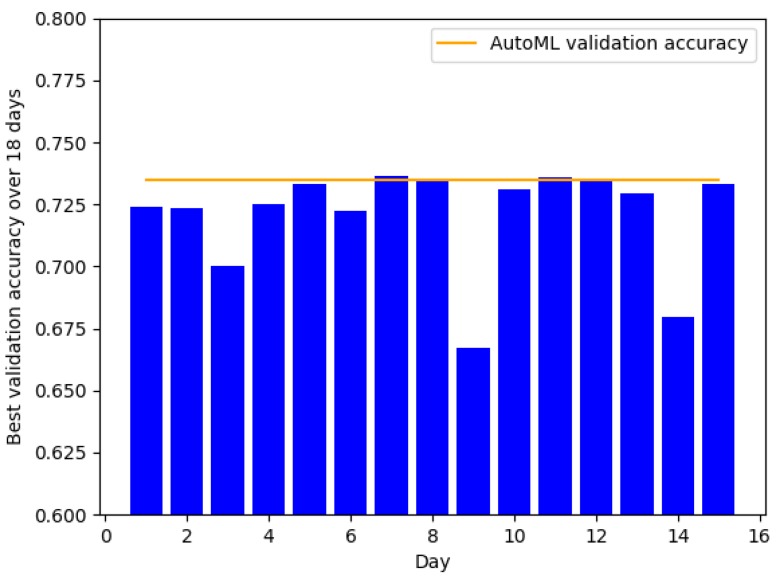
Validation accuracy over 15 days by the graduate student on the Cardiovascular Disease dataset.

In this section, we compare the classification accuracies obtained by the student with that of AutoML. Since the student selects the final model based on the best validation accuracy, this experiment will show how quickly the student can find a good model for a particular dataset. Figure 3 and Figure 4 show the classification over 15–18 day periods for UCI Heart and Cardiovascular Disease datasets, respectively. For both datasets, AutoML achieves competitive validation accuracies compared to that of the student. On the UCI-Heart dataset, the student was able to find a good model from the first day. Since this dataset only has a small number of samples (303 in total), simple classifiers such as linear support vector machine and logistic regression tend to work well. Moreover, the small dataset size makes it faster to run an algorithm and thus reduce the overall development time. On the Cardiovascular Disease dataset, it took the student significantly longer time (seven days) to find a good model. This could be because the second dataset is more complex, demonstrated by the lower validation accuracy of linear classifiers compared to the previous dataset. Moreover, the number of data points is also significantly larger (70,000 of Cardiovascular Disease vs. 303 of UCI-Heart). Our experiment suggests that the time-saving factor is larger for more complex datasets when using AutoML instead of manual model search.

### 3.2. Comparison of AutoML’s and Graduate Student’s Test-Set Performances

Once the final models are selected for the two datasets based on the best validation accuracies, the student performed inference on the test sets to obtain the final performance measures. Table 3 and Table 4 compare the final models’ classification accuracy, area under ROC curve, and area under precision recall curve to that of AutoML models. Note that AutoML models were evaluated on exactly the same test sets to make the results comparable. AutoML achieves slightly better mean accuracy for the UCI-Heart dataset, and similar accuracy for the Cardiovascular Disease dataset compared to the student. In addition, AutoML achieves significantly better areas under curves on both datasets. This suggests that AutoML classifiers generalize much better than their manual counterparts. Most importantly, AutoML only takes 30 min to build a competitive classifier for each dataset, compared to long periods of time (432 h for UCI and 360 h for Cardiovascular datasets) taken by the graduate student to develop similar classifiers.

Other state-of-the-art studies on the Heart UCI dataset have shown results comparable with those of the graduate student and AutoML. Table 5 presents performance accuracies from training different machine learning models on the Heart UCI dataset. The accuracies obtained by the student and AutoML are on par with those reported in the recent literature. This further supports the claim that the AutoML method is able to quickly find competitive classifiers with minimal human effort.

The Heart UCI dataset contains 76 features, but only 13 most-important features are included since most studies and published papers utilize them to build machine learning models on. This makes it possible to compare our results to these published papers, in order to serve as the baseline to check if the results obtained by the Graduate student and Auto-sklearn are competent enough. However, the potential downside to reduced feature space is loss of information. The other features (not included within these 13 attributes) include information on the subject’s response to exercise Electrocardiogram and cigarette smoking habits among others [38].

## 4. Conclusions

This study intends to propose the use of AutoML for adoption in the clinical domain by breaking the perception that machine learning is accessible to trained experts only. For the first time, we evaluate the performance of an AutoML library (Auto-Sklearn) on two cardiovascular disease datasets and compare the results to that obtained by a graduate student after a month of effort in training multiple classifiers on the datasets. These two cardiovascular datasets contain clinical data from trial subjects and whether or not they have cardiovascular disease, so that, given a new subject’s data, the model (learned patterns from given data) can predict the presence or absence of cardiovascular disease with a reasonably good accuracy. The results indicate that the graduate student and AutoML report similar accuracies on the two datasets, on par with other state-of-the-art studies. The area under curves for AutoML is significantly higher indicating that the model built by AutoML generalizes better than that of the graduate student. In addition, the time taken by AutoML to produce these results is just around 30 min per dataset, which is significantly less compared to about 400 h taken by the graduate student. The number of lines of code for AutoML is also significantly lesser compared to the several hundred code lines used by the graduate student, hence justifying the ease of use. Thus, our experimental results strongly suggest that AutoML is a promising approach that enables non-technical users to quickly build competitive machine learning models that work as well as those designed by humans with experience in machine learning. This finding is expected to change the way biomedical researchers and physicians view machine learning. The development of AutoML technology is likely to make machine learning tools more accessible and speed up the research discovery process in the clinical community. Although this study focuses on cardiovascular disease datasets, we conjecture that the key findings related to the efficiency and efficacy of AutoML will hold for other biomedical datasets. In the future, we will investigate the effects of AutoML on other clinically relevant tasks such as tumor detection and segmentation from medical images. Another important advantage of AutoML techniques is that they can incorporate additional constraints when searching for AI models. For example, physicians might want to maximize the classification accuracy while ensuring that the classifier’s sensitivity is higher than a certain threshold. Such constraints are hard to optimize in the traditional AI framework. Our future work will evaluate this complex scenario. We expect that the advantage of AutoML will be more prominent when the complexity of the task increases.

## Figures and Tables

**Table 1:** Thirteen attributes of the Heart UCI (University of California, Irvine, CA, USA) dataset.

Attribute	Type	Description
Age	Continuous	Age in years
Cp	Discrete	Chest pain type (4 values)
Trestbps	Continuous	Resting blood pressure (in mm Hg on admission to the hospital)
Chol	Continuous	Serum cholestoral in mg/dL
Fbs	Discrete	Fasting blood sugar > 120 mg/dL 1 = true; 0 = false
Restecg	Discrete	Resting electrocardiographic results (values 0,1,2)
Thalach	Continuous	Maximum heart rate achieved
Exang	Discrete	Exercise induced angina (1 = yes; 0 = no)
Oldpeak	Continuous	ST depression induced by exercise relative to rest
Slope	Discrete	The slope of the peak Exercise ST segment (values 0,1,2)
Ca	Discrete	Number of major vessels (0–4) colored by flourosopy
Thal	Discrete	Nature of defect, values (0–3)
Target	Discrete	Presence or absence of heart disease, values (1,0)

**Table 2:** Twelve attributes of Cardiovascular Diseases dataset.

Attribute	Type	Description
Age	Continuous	Age of the patient in days
Gender	Discrete	1: women, 2: men
Height (cm)	Continuous	Height of the patient in cm
Weight (kg)	Continuous	Weight of the patient in kg
Ap_hi	Continuous	Systolic blood pressure
Ap_lo	Continuous	Diastolic blood pressure
Cholesterol	Discrete	1: normal, 2: above normal, 3: well above normal
Gluc	Discrete	1: normal, 2: above normal, 3: well above normal
Smoke	Discrete	whether patient smokes or not
Alco	Discrete	Alcohol intake-Binary feature
Active	Discrete	Physical activity-Binary feature
Cardio	Discrete	Presence or absence of cardiovascular disease

**Table 3:** Comparison of AutoML and the graduate student’s classification performances and total time on UCI test set.

	Accuracy	AUC-ROC	AUC-PR	Total Ttime (h)
**Graduate student**	0.84	0.82	0.80	432
**AutoML**	0.85	0.93	0.94	0.5

**Table 4:** Comparison of AutoML and graduate student’s classification performances and total time on the Cardiovascular test set.

	Accuracy	AUC-ROC	AUC-PR	Total Time (h)
**Graduate student**	0.74	0.73	0.68	360
**AutoML**	0.74	0.8	0.79	0.5

**Table 5:** Accuracies reported by previous studies on the Heart UCI Dataset compared to accuracies of the graduate student and AutoML.

Author	Reported Accuracy
Shouman et al. [18]	0.841
Duch et al. [35]	0.856
Wang et al. [36]	0.8337
Srinivas et al. [19]	0.837
Tomar and Agarwal [20]	0.8559
Graduate student (this paper)	0.84
AutoML (this paper)	0.85

## Supplementary Materials

The following are available online at https://www.mdpi.com/2077-0383/8/7/1050/s1, Table S1: Validation performances of different algorithms on Heart UCI dataset over days, Table S2: Validation performances of different algorithms on Cardiovascular disease dataset over days, Table S3: Descriptions and parameter settings of algorithms used by the graduate student on the datasets.

## Abbreviations

The following abbreviations are used in this manuscript:

AI	Artificial Intelligence
AutoML	Auto Machine Learning
UCI	University of California, Irvine
GPU	Graphic Processing Unit
TPU	Tensor Processing Unit
ROC	Reciever Operating Characteristic
PR	Precision–Recall
PCA	Principal Component Analysis
