# Supplementary material for: Physician-Friendly Machine Learning: A Case Study with Cardiovascular Disease Risk Prediction
Source: J Clin Med. 2019 Jul 18;8(7):1050. doi: 10.3390/jcm8071050 (PMC6678298; doi:10.3390/jcm8071050)
Supplement: Supplementary file 1 [file jcm-08-01050-s001.zip › jcm-540468-Supplementary/Table S1.pdf]

Validation accuracy and areas under curves on UCI Heart dataset over different days				
	**Algorithm**	**Validation accuracy**	**Area under PR**	**Area under ROC**
1	Logistic regression with default parameters	0.78878	0.9011128	0.89478
	Linear SVM with default parameters	0.84756	0.896318	0.895693
	SVM with RBF kernel with default parameters	0.808	0.891344	0.88138
2	Logistic Regression (with default parameters) run on best feature subset selected using F-test	0.8128	0.8880569	0.88159
	Logistic Regression (with default parameters) run on best feature subset selected using mutual information test	0.808048	0.8975	0.89674
	Linear SVM (with default parameters) run on best feature subset selected using F-test	0.84756	0.896318	0.895693
	Linear SVM (with default parameters) run on best feature subset selected using mutual information test	0.84756	0.896318	0.895693
	SVM with RBF kernel (with default parameters) run on best feature subset selected using F-test	0.8329	0.904811	0.89211665
	SVM with RBF kernel (with default parameters) run on best feature subset selected using mutual information test	0.823	0.903723	0.8940077
3	Hyperparameter selected SVM (with RBF kernel)	0.813	0.886121	0.88038
	Decision Tree classifier with default parameters	0.699878	0.66709	0.697596
	Random forest with default parameters	0.783536	0.8882388	0.8793
	Logistic Regression (with default parameters) run on best feature subset selected using RFE	0.8129	0.890859	0.894463

4	Linar SVM (with default parameters) run on best feature subset selected using RFE	0.84756	0.896318	0.895693
	Hyperparameter selected SVM (with RBF kernel) run on best feature subset selected using F-test	0.8329	0.904811	0.89211
	Hyperparameter selected SVM (with RBF kernel) run on best feature subset selected using mutual information test	0.82292	0.904803	0.89203
	Hyperparameter selected decision tree	0.74402	0.745473	0.7643768
	Extra Trees classifier with deafult parameters	0.79841	0.8530859	0.86375
5	Hyperparameter selected Logistic Regression	0.80817	0.89997	0.89817
	Hyperparameter selected Logistic Regression run over best feature subset selected using RFE	0.82256	0.8974	0.89742
	Bagged decision tree with default bagging parameters	0.74939	0.8477148	0.837058
	Hyperparameter selected random forest	0.813048	0.894048	0.88423
	Hyperparameter selected extra trees classifier	0.8328048	0.894655	0.888
6	Hyperparameter selected linear SVM run on best feature subset selected using F-test	0.8278	0.896389	0.896012
	Hyperparameter selected linear SVM run on best feature subset selected using mutual-information-test	0.80792	0.8896	0.88867
	Hyperparameter selected linear SVM run on best feature subset selected using RFE	0.84756	0.89753	0.899134
	K Nearest neighbors run with deafult parameters	0.7639	0.833959	0.8481658
	Hyperparameter selected KNN	0.79829	0.826397	0.8450444
	MLP with default parameters	0.84292	0.894446	0.8930052
7	Hyperparameter selected Linear SVM	0.84756	0.89753	0.89913

8	Hyperparameter selected Linear SVM run on best feature subset selected using the F-test	0.84756	0.89753	0.89913
	Hyperparameter selected Linear SVM run on best feature subset selected using the Mutual information test	0.8228	0.89753	0.89913
	Bagged decision tree with hyperparameter selected bagging parameters	0.74402	0.74547	0.7643768
9	GBT with default parameters	0.754268	0.863732	0.8455228
10	Bagged KNN with default bagging parameters	0.79317	0.8785502	0.8726475
	Bagged decision tree with hyperparameter selected bagging parameters	0.813048	0.85738	0.864297
11	MLP using hold out cross validation dataset to adjust the hidden layer sizes	0.84292	0.894446	0.8930052
12	GBT with early stopping	0.74926	0.8598156	0.84612668
	Adaboost classifier with default parameters	0.758658	0.8153026	0.8051948
13	Hyperparameter selected GBT	0.78829	0.848089	0.84422419
14	Adaboost with SVM (RBF kernel) as base estimator with default boosting parameters	0.813414	0.891518	0.8836409
15	Adaboost with SVM (RBF kernel) as base estimator with hyperparameter selected boosting parameters	0.813414	0.891518	0.8836409
16	Adaboost (with Decision tree as the base estimator) with default boosting parameters	0.749268	0.859368	0.832627
	Adaboost (with Decision tree as the base estimator) with hyperparameter selection of boosting parameters	0.749268	0.859368	0.832627
17	Voting classifier with Logistic Regression and SVM (with RBF kernel) as the base estimators	0.8228048	0.8999173	0.8955798
