# Supplementary material for: Physician-Friendly Machine Learning: A Case Study with Cardiovascular Disease Risk Prediction
Source: J Clin Med. 2019 Jul 18;8(7):1050. doi: 10.3390/jcm8071050 (PMC6678298; doi:10.3390/jcm8071050)
Supplement: Supplementary file 1 [file jcm-08-01050-s001.zip › jcm-540468-Supplementary/Table S2.pdf]

Validation accuracy and areas under curves on Cardiovascular Disease dataset over different days				
**Day**	**Algorithm**	**Validation accuracy**	**Area under PR**	**Area under AUC**
1	Logistic Regression with default parameters	0.723714	0.76851	0.7865167
	Linear SVM with default parameters	0.723785	0.7719484	0.7885721
2	Logistic regression (with default parameters) run on best feature subset selected using mutual_information test	0.723714	0.768510596	0.78651675
	Logistic regression (with default parameters) run on best feature subset selected using F-test	0.723714	0.768510596	0.78651675
3	Decision Trees with default parameters	0.633875	0.584521224	0.6338543
	Random forest with default parameters	0.700071799	0.7220255	0.75192896
4	Logistic regression (with default parameters) run on best feature subset selected using RFE	0.723714	0.768510596	0.78651675
	Hyperparameter selected decision trees	0.72516	0.74735396	0.77891727
	Extra Trees classifier with default parameters	0.6923212	0.714689946	0.747498768
5	Hyperparameter selected logistic regression	0.72216	0.766638	0.785086
	Hyperparameter selected Logistic regression run on best feature subset selected using RFE	0.72216	0.766638	0.785086
	Bagged decision tree with default tree and default bagging parameters	0.69457	0.71740559	0.746457
	Hyperparameter selected random forest	0.733285	0.782507669	0.799203889
	Hyperparameter selected extra trees classifier	0.724499714	0.76900928	0.786646167
6	Hyperparameter selected Logistic regression run on best feature subset selected using F-test	0.72216	0.766638	0.785086
	Hyperparameter selected Logistic regression run on best feature subset selected using mutual-information test	0.721767	0.7667635	0.78516308
	K nearest neighbors (KNN) with default parameters	0.6490004	0.6518057	0.6937506
	Hyperparameter selected KNN	0.6633037	0.695850793	0.725343673
	**Bagged KNN with default bagging parameters and hyperparameter selected KNN (using hold out cross validation)	0.672857143	0.617624076	0.672843752
7	Bagged decision tree with default bagging parameters and hyperparameter selected trees	0.72442867	0.7543878	0.78348809
	Bagged decision tree with Hyperparameter selected bagging parameters and hyperparameter selected trees	0.7365357	0.784403633	0.80085053

8	MLP with default parameters	0.7344998	0.7839049	0.80077518
9	KNN with n_neighbors selected by common practice	0.66307	0.7055441	0.721821787
	KNN with weights parameter 'uniform'	0.663303765	0.69585079	0.72534367
	KNN with weights parameter 'distance'	0.66719669	0.70235477	0.72515836
10	MLP trained with hidden layer sizes selected using common practice	0.730107	0.776824243	0.794522289
	Gradient Boosted Trees (GBT) with default parameters	0.73574998	0.786339793	0.802404022
11	**Using hold out cross validation dataset to adjust the hidden layer sizes in MLP	0.728571429	0.672313798	0.7285437
	GBT with hyperparameter search	0.73551789	0.785422854	0.801896237
12	Adaboost classifier with default parameters	0.7294466	0.7746921	0.79510308
13	Adaboost with decision tree as the base classifier	0.6798037	0.72085929	0.73191027
14	Adaboost with decision tree as the base classifier and hyperparameter selected of boosting parameters	0.733339338	0.779142077	0.797869636
	Voting classifier with Logistic Regression and Random Forest classifiers as base estimators	0.732178	0.779432729	0.7968886
15	**SVM with RBF Kernel (with default parameters) with PCA applied input data	0.735267857	0.6771019	0.735248314
	**SVM with Linear Kernel (with default parameters) with PCA applied input data	0.725803571	0.674904287	0.725755039
	Voting classifier with gradient Boosted trees and Adaboost classifier as the base estimators	0.73560714	0.785655199	0.802340698
