# Supplementary material for: Physician-Friendly Machine Learning: A Case Study with Cardiovascular Disease Risk Prediction
Source: J Clin Med. 2019 Jul 18;8(7):1050. doi: 10.3390/jcm8071050 (PMC6678298; doi:10.3390/jcm8071050)
Supplement: Supplementary file 1 [file jcm-08-01050-s001.zip › jcm-540468-Supplementary/Table S3.pdf]

Dataset-- Heart UCI

Training data size= 203 samples (108 1s and 95 0s)

Test data size = 100 samples (57 1s AND 43 0s)

k-fold cross validation on training data with k=5

**Algorithm**	**Description**	**Package**	**Parameters**
Logistic Regression	Logistic Regression with default parameters	sklearn.linear_model.LogisticRegression	LogisticRegression(self, penalty='l2', dual=False, tol=1e-4, C=1.0, fit_intercept=True, intercept_scaling=1, class_weight=None, random_state=None, solver='warn', max_iter=100, multi_class='warn', verbose=0, warm_start=False, n_jobs=None)
	F-classif feature selection technique applied to select best feature subset (or k) by plotting k versus validation accuracies. Logistic regression with best feature subset and default parameters is applied to data	sklearn.feature_selection.SelectKBest<br><br>sklearn.linear_model.LogisticRegression	SelectKBest(self, score_func=f_classif, k=10)<br><br>LogisticRegression(self, penalty='l2', dual=False, tol=1e-4, C=1.0, fit_intercept=True, intercept_scaling=1, class_weight=None, random_state=None, solver='warn', max_iter=100, multi_class='warn', verbose=0, warm_start=False, n_jobs=None)
	mutual_info_classif feature selection technique applied to select best feature subset (or k) by plotting k versus validation accuracies.<br>Logistic regression with best feature subset and	sklearn.feature_selection.SelectKBest<br><br>sklearn.linear_model.LogisticRegression	SelectKBest(self, score_func=mutual_info_classif, k=12)<br><br>LogisticRegression(self, penalty='l2', dual=False, tol=1e-4, C=1.0, fit_intercept=True, intercept_scaling=1,

	default parameters is applied to data		class_weight=None, random_state=None, solver='warn', max_iter=100, multi_class='warn', verbose=0, warm_start=False, n_jobs=None)
	Recursive Feature Elimination (RFE) feature selection technique applied to select best feature subset by plotting subset size versus validation accuracies.<br>Logistic regression with best feature subset and default parameters is applied to data	sklearn.feature_selection.RFE<br><br>sklearn.linear_model.LogisticRegression	RFE(self, estimator, n_features_to_select=12, step=1, verbose=0)<br><br>LogisticRegression(self, penalty='l2', dual=False, tol=1e-4, C=1.0, fit_intercept=True, intercept_scaling=1, class_weight=None, random_state=None, solver='warn', max_iter=100, multi_class='warn', verbose=0, warm_start=False, n_jobs=None)
	Hyperparameter tuned logistic regression	sklearn.linear_model.LogisticRegression<br><br>sklearn.model_selection.validation_curve	LogisticRegression(self, penalty='l2', dual=False, tol=1e-4, C=3.0, fit_intercept=True, intercept_scaling=1, class_weight=None, random_state=None, solver='warn', max_iter=15, multi_class='warn', verbose=0, warm_start=False, n_jobs=None)
	Best hyperparameters applied to logistic regression with f-classif.	sklearn.feature_selection.SelectKBest<br><br>sklearn.linear_model.LogisticRegression	SelectKBest(self, score_func=f_classif, k=13)<br><br>LogisticRegression(self, penalty='l2', dual=False, tol=1e-4, C=3.0, fit_intercept=True, intercept_scaling=1,

			class_weight=None, random_state=None, solver='warn', max_iter=15, multi_class='warn', verbose=0, warm_start=False, n_jobs=None)
	Best hyperparameters applied to logistic regression with mutual_info_classif.	sklearn.feature_selection.SelectKBest<br><br>sklearn.linear_model.LogisticRegression	SelectKBest(self, score_func=mutual_info classif, k=12)<br><br>LogisticRegression(self, penalty='l2', dual=False, tol=1e-4, C=3.0, fit_intercept=True, intercept_scaling=1, class_weight=None, random_state=None, solver='warn', max_iter=15, multi_class='warn', verbose=0, warm_start=False, n_jobs=None)
	Best hyperparameters applied to logistic regression with RFE.	sklearn.feature_selection.RFE<br><br>sklearn.linear_model.LogisticRegression	RFE(self, estimator, n_features_to_select=11, step=1, verbose=0)<br><br>LogisticRegression(self, penalty='l2', dual=False, tol=1e-4, C=3.0, fit_intercept=True, intercept_scaling=1, class_weight=None, random_state=None, solver='warn', max_iter=15, multi_class='warn', verbose=0, warm_start=False, n_jobs=None)
Linear Support Vector Classifier	SVC with Linear kernel with default parameters	sklearn.svm.SVC	SVC(self, C=1.0, kernel='linear', degree=3, gamma='auto_deprecated', coef0=0.0,

			shrinking=True,<br>probability=False,<br>tol=1e-3,<br>cache_size=200,<br>class_weight=None,<br>verbose=False,<br>max_iter=-1,<br>decision_function_shape='ovr',<br>random_state=None)
	F-classif feature selection technique applied to select best feature subset (or k) by plotting k versus validation accuracies.<br>Linear SVC with best feature subset and default parameters is applied to data	sklearn.feature_selection.SelectKBest<br><br>sklearn.svm.SVC	SelectKBest(self, score_func=f_classif, k=19)<br><br>SVC(self, C=1.0, kernel='linear', degree=3, gamma='auto_deprecated', coef0=0.0, shrinking=True, probability=False, tol=1e-3, cache_size=200, class_weight=None, verbose=False, max_iter=-1, decision_function_shape='ovr', random_state=None)
	mutual_info_classif feature selection technique applied to select best feature subset (or k) by plotting k versus validation accuracies.<br>Linear SVC with best feature subset and default parameters is applied to data	sklearn.feature_selection.SelectKBest<br><br>sklearn.linear_model.LogisticRegression	SelectKBest(self, score_func=f_classif, k=17)<br><br>SVC(self, C=1.0, kernel='linear', degree=3, gamma='auto_deprecated', coef0=0.0, shrinking=True, probability=False, tol=1e-3, cache_size=200, class_weight=None, verbose=False, max_iter=-1, decision_function_shape

			='ovr', random_state=None)
	Recursive Feature Elimination (RFE) feature selection technique applied to select best feature subset by plotting subset size versus validation accuracies.<br>Linear SVC with best feature subset and default parameters is applied to data	sklearn.feature_selection.RFE<br><br>sklearn.svm.SVC	RFE(self, estimator, n_features_to_select=16, step=1, verbose=0)<br><br>SVC(self, C=1.0, kernel='linear', degree=3, gamma='auto_deprecated', coef0=0.0, shrinking=True, probability=False, tol=1e-3, cache_size=200, class_weight=None, verbose=False, max_iter=-1, decision_function_shape='ovr', random_state=None)
	Hyperparameter tuned Linear SVC	sklearn.svm.SVC<br><br>sklearn.model_selection.validation_curve	SVC(C=1.2, cache_size=200, class_weight=None, coef0=0.0, decision_function_shape='ovr', degree=3, gamma='auto_deprecated', kernel='linear', max_iter=600, probability=1, random_state=None, shrinking=True, tol=0.001, verbose=False)
	Best hyperparameters applied to linear SVC with f-classif.	sklearn.feature_selection.SelectKBest<br><br>sklearn.svm.SVC	SelectKBest(self, score_func=f_classif, k=19)<br><br>SVC(C=1.2, cache_size=200, class_weight=None, coef0=0.0, decision_function_shape='ovr', degree=3, gamma='auto_deprecated', kernel='linear',

			max_iter=600,<br>probability=1,<br>random_state=None,<br>shrinking=True,<br>tol=0.001,<br>verbose=False)
	Best hyperparameters applied to linear SVC with mutual_info_classif.	sklearn.feature_selection.SelectKBest<br><br>sklearn.svm.SVC	SelectKBest(self, score_func=mutual_info_classif, k=19)<br><br>SVC(C=1.2, cache_size=200, class_weight=None, coef0=0.0, decision_function_shape='ovr', degree=3, gamma='auto_deprecated', kernel='linear', max_iter=600, probability=1, random_state=None, shrinking=True, tol=0.001, verbose=False)
	Best hyperparameters applied to linear SVC with RFE.	sklearn.feature_selection.RFE<br><br>sklearn.svm.SVC	RFE(self, estimator, n_features_to_select=18, step=1, verbose=0)<br><br>SVC(self, C=1.2, kernel='linear', degree=3, gamma='auto_deprecated', coef0=0.0, shrinking=True, probability=False, tol=1e-3, cache_size=200, class_weight=None, verbose=False, max_iter=600, decision_function_shape='ovr', random_state=None)
SVC with RBF Kernel	SVC with RBF kernel with default parameters	sklearn.svm.SVC	SVC(self, C=1.0, kernel='rbf', degree=3, gamma='auto_deprecate

			d', coef0=0.0, shrinking=True, probability=False, tol=1e-3, cache_size=200, class_weight=None, verbose=False, max_iter=-1, decision_function_shape='ovr', random_state=None)
	F-classif feature selection technique applied to select best feature subset (or k) by plotting k versus validation accuracies. SVC (with RBF kernel) with best feature subset and default parameters is applied to data	sklearn.feature_selection.SelectKBest<br><br>sklearn.svm.SVC	SelectKBest(self, score_func=f_classif, k=10)<br><br>SVC(self, C=1.0, kernel='rbf', degree=3, gamma='auto_deprecated', coef0=0.0, shrinking=True, probability=False, tol=1e-3, cache_size=200, class_weight=None, verbose=False, max_iter=-1, decision_function_shape='ovr', random_state=None)
	mutual_info_classif feature selection technique applied to select best feature subset (or k) by plotting k versus validation accuracies. SVC(with RBF kernel) with best feature subset and default parameters is applied to data	sklearn.feature_selection.SelectKBest<br><br>sklearn.svm. SVC	SelectKBest(self, score_func=mutual_info_classif, k=10)<br><br>SVC(self, C=1.0, kernel='rbf', degree=3, gamma='auto_deprecated', coef0=0.0, shrinking=True, probability=False, tol=1e-3, cache_size=200, class_weight=None, verbose=False, max_iter=-1, decision_function_shape

			='ovr', random_state=None)
	Hyperparameter tuned SVC with RBF kernel	sklearn.svm.SVC<br><br>sklearn.model_selection.validation_curve	SVC(self, C=1.0, kernel='rbf', degree=3, gamma= 0.1, coef0=0.0, shrinking=True, probability=False, tol=1e-3, cache_size=200, class_weight=None, verbose=False, max_iter=300, decision_function_shape='ovr', random_state=None)
	Best hyperparameters applied to SVC (RBF kernel) with f-classif.	sklearn.feature_selection.SelectKBest<br><br>sklearn.svm.SVC	SelectKBest(self, score_func=f_classif, k=13)<br><br>SVC(self, C=1.0, kernel='rbf', degree=3, gamma= 0.1, coef0=0.0, shrinking=True, probability=False, tol=1e-3, cache_size=200, class_weight=None, verbose=False, max_iter=300, decision_function_shape='ovr', random_state=None)
	Best hyperparameters applied to SVC (RBF kernel) with mutual_info_classif.	sklearn.feature_selection.SelectKBest<br><br>sklearn.svm.SVC	SelectKBest(self, score_func=mutual_info_classif, k=10)<br><br>SVC(self, C=1.0, kernel='rbf', degree=3, gamma= 0.1, coef0=0.0, shrinking=True, probability=False, tol=1e-3, cache_size=200, class_weight=None, verbose=False, max_iter=300, decision_function_shape

			='ovr', random_state=None)
Decision Tree classifier	Decision Tree classifier with default parameters	sklearn.tree.DecisionTreeClassifier	DecisionTreeClassifier(self, criterion="gini", splitter="best", max_depth=None, min_samples_split=2, min_samples_leaf=1, min_weight_fraction_leaf=0., max_features=None, random_state=None, max_leaf_nodes=None, min_impurity_decrease=0., min_impurity_split=None, class_weight=None, presort=False)
	Hyperparameter selected decision tree	sklearn.model_selection.validation_curve<br><br>sklearn.tree.DecisionTreeClassifier	DecisionTreeClassifier(class_weight=None, criterion='gini', max_depth=3, max_features=None, max_leaf_nodes=None, min_impurity_decrease=0.0,min_impurity_split=None, min_samples_leaf=1, min_samples_split=2, min_weight_fraction_leaf=0.0, presort=False, random_state=None, splitter='best')
	Bagged decision tree with default tree and default bagging parameters	sklearn.ensemble.BaggingClassifier<br><br>sklearn.tree.DecisionTreeClassifier	BaggingClassifier(base_estimator=DecisionTreeClassifier(class_weight=None, criterion='gini', max_depth=None, max_features=None, max_leaf_nodes=None, min_impurity_decrease=0.0, min_impurity_split=None, min_samples_leaf=1, min_samples_split=2,

			min_weight_fraction_leaf=0.0, presort=False, random_state=None,splitter='best'),bootstrap=True,bootstrap_features=False, max_features=1.0, max_samples=1.0, n_estimators=10,n_jobs=None,oob_score=False,random_state=None, verbose=0,warm_start=False)
	Bagged decision tree with default bagging parameters and hyperparameter selected decision tree	sklearn.ensemble.BaggingClassifier<br><br>sklearn.tree.DecisionTreeClassifier	BaggingClassifier(base_estimator=DecisionTreeClassifier(class_weight=None, criterion='gini', max_depth=3, max_features=None, max_leaf_nodes=None, min_impurity_decrease=0.0,min_impurity_split=None, min_samples_leaf=1, min_samples_split=2, min_weight_fraction_leaf=0.0, presort=False, random_state=None,splitter='best'),bootstrap=True,bootstrap_features=False, max_features=1.0, max_samples=1.0, n_estimators=10, n_jobs=None, oob_score=False, random_state=None, verbose=0, warm_start=False)
	Bagged decision tree with hyperparameter selected bagging parameters and hyperparameter selected Decision Tree	sklearn.ensemble.BaggingClassifier<br><br>sklearn.tree.DecisionTreeClassifier	BaggingClassifier(base_estimator=DecisionTreeClassifier(class_weight=None, criterion='gini', max_depth=3, max_features=None, max_leaf_nodes=None, min_impurity_decrease

			=0.0,min_impurity_split =None, min_samples_leaf=1, min_samples_split=2, min_weight_fraction_le af=0.0, presort=False, random_state=None, splitter='best'), bootstrap=True, bootstrap_features=Fals e, max_features=7, max_samples=0.8, n_estimators=10, n_jobs=None, oob_score=False, random_state=None, verbose=0, warm_start=False)
Random Forest Classifier	Random forest classifier with default parameters	sklearn.ensemble.Rando mForestClassifier	RandomForestClassifier (bootstrap=True, class_weight=None, criterion='gini', max_depth=None, max_features='auto', max_leaf_nodes=None, min_impurity_decrease =0.0, min_impurity_split=No ne, min_samples_leaf=1, min_samples_split=2, min_weight_fraction_le af=0.0, n_estimators='warn', n_jobs=None, oob_score=False, random_state=None, verbose=0, warm_start=False)
	Hyperparameter selected random forest classifier	sklearn.model_selection .validation_curve<br><br>sklearn.ensemble.Rando mForestClassifier	RandomForestClassifier (bootstrap=True, class_weight=None, criterion='gini', max_depth=3, max_features='auto', max_leaf_nodes=None,

			min_impurity_decrease =0.0, min_impurity_split=None,min_samples_leaf=1, min_samples_split=2, min_weight_fraction_leaf=0.0,n_estimators=55, n_jobs=None, oob_score=False, random_state=None, verbose=0, warm_start=False)
Extra Trees classifier	Extra Trees classifier with default parameters	sklearn.ensemble import ExtraTreesClassifier	ExtraTreesClassifier(bootstrap=False, class_weight=None, criterion='gini', max_depth=None, max_features='auto', max_leaf_nodes=None, min_impurity_decrease =0.0, min_impurity_split=None, min_samples_leaf=1, min_samples_split=2, min_weight_fraction_leaf=0.0, n_estimators='warn', n_jobs=None, oob_score=False, random_state=None, verbose=0, warm_start=False)
	Extra trees classifier with hyperparameter selection	sklearn.model_selection .validation_curve<br><br>sklearn.ensemble import ExtraTreesClassifier	ExtraTreesClassifier(bootstrap=False, class_weight=None, criterion='gini', max_depth=5, max_features='auto', max_leaf_nodes=None, min_impurity_decrease =0.0, min_impurity_split=None, min_samples_leaf=1, min_samples_split=2,

			min_weight_fraction_leaf=0.0, n_estimators=55, n_jobs=None, oob_score=False, random_state=None, verbose=0, warm_start=False)
K nearest neighbors classifier	KNN classifier with default parameters	sklearn.neighbors. KNeighborsClassifier	KNeighborsClassifier(algorithm='auto', leaf_size=30, metric='minkowski', metric_params=None, n_jobs=None, n_neighbors=5, p=2, weights='uniform')
	Hyperparameter selected KNN	sklearn.model_selection .validation_curve<br><br>sklearn.neighbors. KNeighborsClassifier	KNeighborsClassifier(algorithm='auto', leaf_size=30, metric='minkowski', metric_params=None, n_jobs=None, n_neighbors=3, p=1, weights='uniform')
	Bagged hyperparameter selected KNN	sklearn.ensemble.BaggingClassifier<br><br>sklearn.neighbors. KNeighborsClassifier	BaggingClassifier(base_estimator=KNeighborsClassifier(algorithm='auto', leaf_size=30, metric='minkowski', metric_params=None, n_jobs=None, n_neighbors=3, p=1, weights='uniform'), bootstrap=True, bootstrap_features=False, max_features=1.0, max_samples=1.0, n_estimators=10, n_jobs=None, oob_score=False, random_state=None, verbose=0, warm_start=False)
MLP Classifier	MLP with default parameters	sklearn.neural_network. MLPClassifier	MLPClassifier(activation='relu', alpha=0.0001, batch_size='auto', beta_1=0.9,

			beta_2=0.999, early_stopping=False, epsilon=1e-08, hidden_layer_sizes=(100,), learning_rate='constant', learning_rate_init=0.001, max_iter=200, momentum=0.9, n_iter_no_change=10, nesterovs_momentum=True, power_t=0.5, random_state=None, shuffle=True, solver='adam', tol=0.0001, validation_fraction=0.1, verbose=False, warm_start=False)
	Using hold out cross validation dataset to adjust the hidden layer sizes and max_iterations in MLP	sklearn.neural_network.MLPClassifier	MLPClassifier(activation='relu', alpha=0.0001, batch_size='auto', beta_1=0.9, beta_2=0.999, early_stopping=False, epsilon=1e-08, hidden_layer_sizes=(100,), learning_rate='constant', learning_rate_init=0.001, max_iter=200, momentum=0.9, n_iter_no_change=10, nesterovs_momentum=True, power_t=0.5, random_state=None, shuffle=True, solver='adam', tol=0.0001, validation_fraction=0.1, verbose=False, warm_start=False)
Gradient Boosted Trees (GBT)	GBT with default parameters	sklearn.ensemble.GradientBoostingClassifier	GradientBoostingClassifier(criterion='friedman_mse', init=None, learning_rate=0.1,

			loss='deviance', max_depth=3, max_features=None, max_leaf_nodes=None, min_impurity_decrease=0.0, min_impurity_split=None, min_samples_leaf=1, min_samples_split=2, min_weight_fraction_leaf=0.0, n_estimators=100, n_iter_no_change=None, presort='auto', random_state=None, subsample=1.0, tol=0.0001, validation_fraction=0.1, verbose=0, warm_start=False)
	GBT with early stopping (to counter overfitting)	sklearn.ensemble.GradientBoostingClassifier	GradientBoostingClassifier(criterion='friedman_mse', init=None, learning_rate=0.1, loss='deviance', max_depth=3, max_features=None, max_leaf_nodes=None, min_impurity_decrease=0.0, min_impurity_split=None, min_samples_leaf=1, min_samples_split=2, min_weight_fraction_leaf=0.0, n_estimators=100, n_iter_no_change=5, presort='auto', random_state=None, subsample=1.0, tol=0.0001, validation_fraction=0.2, verbose=0, warm_start=False)

	GBT with hyperparameter search	sklearn.model_selection.validation_curve<br><br>sklearn.ensemble.GradientBoostingClassifier	GradientBoostingClassifier(criterion='friedman_mse', init=None, learning_rate=0.1, loss='deviance', max_depth=3, max_features=None, max_leaf_nodes=None, min_impurity_decrease=0.0, min_impurity_split=None, min_samples_leaf=1, min_samples_split=2, min_weight_fraction_leaf=0.0, n_estimators=37, n_iter_no_change=None, presort='auto', random_state=None, subsample=1.0, tol=0.0001, validation_fraction=0.1, verbose=0, warm_start=False)
Adaboost classifier	Adaboost classifier with default parameters	sklearn.ensemble.AdaBoostClassifier	AdaBoostClassifier(algorithm='SAMME.R', base_estimator=None, learning_rate=1.0, n_estimators=50, random_state=None)
	Boosting RBF SVM with Adaboost	sklearn.ensemble.AdaBoostClassifier	AdaBoostClassifier(algorithm='SAMME.R', base_estimator=SVC(C=1.0, cache_size=200, class_weight=None, coef0=0.0, decision_function_shape='ovr', degree=3, gamma=0.1, kernel='rbf', max_iter=300, probability=1, random_state=None, shrinking=True, tol=0.001, verbose=False),

			learning_rate=1.0, n_estimators=50, random_state=None)
	Boosting decision trees with AdaBoost	sklearn.ensemble.AdaBoostClassifier	AdaBoostClassifier(algorithm='SAMME.R', base_estimator=DecisionTreeClassifier(class_weight=None, criterion='gini', max_depth=3, max_features=None, max_leaf_nodes=None, min_impurity_decrease=0.0, min_impurity_split=None,min_samples_leaf=1, min_samples_split=2, min_weight_fraction_leaf=0.0, presort=False, random_state=None, splitter='best'), learning_rate=1.0, n_estimators=50, random_state=None)
Voting classifier	Voting classifier with the Logistic Regression and Support vector classifier as estimators	sklearn.ensemble.VotingClassifier<br><br>sklearn.linear_model.LogisticRegression<br><br>sklearn.svm.SVC	VotingClassifier(estimators=[('logistic', LogisticRegression(C=3, class_weight=None, dual=False, fit_intercept=True, intercept_scaling=1, max_iter=15, multi_class='warn', n_jobs=None, penalty='l2', random_state=None, solver='warn', tol=0.0001, verbose=0, warm_start=False)), ('svm', SVC(...bf', max_iter=100, probability=1, random_state=None, shrinking=True, tol=0.001, verbose=False))],

			flatten_transform=None, n_jobs=None, voting='soft', weights=None)

Cardiovascular disease dataset

56,000 training samples (27983 1s and 28017 0s)
14,000 testing samples (6996 1s and 7004 0s)

k-fold cross validation (with k=10) used and when computational complexity is high (too much time for evaluation), hold-out cross validation is used.

**Algorithm**	**Description**	**Package**	**Parameters**
Logistic Regression	Logistic Regression with default parameters	sklearn.linear_model.LogisticRegression	LogisticRegression(self, penalty='l2', dual=False, tol=1e-4, C=1.0, fit_intercept=True, intercept_scaling=1, class_weight=None, random_state=None, solver='warn', max_iter=100, multi_class='warn', verbose=0, warm_start=False, n_jobs=None)
	F-classif feature selection technique applied to select best feature subset (or k) by plotting k versus validation accuracies. Logistic regression with best feature subset and default parameters is applied to data	sklearn.feature_selection.SelectKBest<br><br>sklearn.linear_model.LogisticRegression	SelectKBest(self, score_func=f_classif, k=11)<br><br>LogisticRegression(self, penalty='l2', dual=False, tol=1e-4, C=1.0, fit_intercept=True, intercept_scaling=1, class_weight=None, random_state=None, solver='warn', max_iter=100, multi_class='warn', verbose=0, warm_start=False, n_jobs=None)

	mutual_info_classif feature selection technique applied to select best feature subset (or k) by plotting k versus validation accuracies.<br>Logistic regression with best feature subset and default parameters is applied to data	sklearn.feature_selection.SelectKBest<br><br>sklearn.linear_model.LogisticRegression	SelectKBest(self, score_func=mutual_info_classif, k=11)<br><br>LogisticRegression(self, penalty='l2', dual=False, tol=1e-4, C=1.0, fit_intercept=True, intercept_scaling=1, class_weight=None, random_state=None, solver='warn', max_iter=100, multi_class='warn', verbose=0, warm_start=False, n_jobs=None)
	Recursive Feature Elimination (RFE) feature selection technique applied to select best feature subset by plotting subset size versus validation accuracies.<br>Logistic regression with best feature subset and default parameters is applied to data	sklearn.feature_selection.RFE<br><br>sklearn.linear_model.LogisticRegression	RFE(self, estimator, n_features_to_select=11, step=1, verbose=0)<br><br>LogisticRegression(self, penalty='l2', dual=False, tol=1e-4, C=1.0, fit_intercept=True, intercept_scaling=1, class_weight=None, random_state=None, solver='warn', max_iter=100, multi_class='warn', verbose=0, warm_start=False, n_jobs=None)
	Hyperparameter tuned logistic regression	sklearn.linear_model.LogisticRegression<br><br>sklearn.model_selection.validation_curve	LogisticRegression(C=0.2, class_weight=None, dual=False, fit_intercept=True, intercept_scaling=1, max_iter=7, multi_class='warn', n_jobs=None, penalty='l2', random_state=None, solver='warn', tol=0.0001,

			verbose=0,<br>warm_start=False)
	Best hyperparameters applied to logistic regression with f-classif.	sklearn.feature_selection.SelectKBest<br><br>sklearn.linear_model.LogisticRegression	SelectKBest(self, score_func=f_classif, k=11)<br><br>LogisticRegression(C=0.2, class_weight=None, dual=False, fit_intercept=True, intercept_scaling=1, max_iter=7, multi_class='warn', n_jobs=None, penalty='l2', random_state=None, solver='warn', tol=0.0001, verbose=0, warm_start=False)
	Best hyperparameters applied to logistic regression with mutual_info_classif.	sklearn.feature_selection.SelectKBest<br><br>sklearn.linear_model.LogisticRegression	SelectKBest(self, score_func=mutual_info classif, k=10)<br><br>LogisticRegression(C=0.2, class_weight=None, dual=False, fit_intercept=True, intercept_scaling=1, max_iter=7, multi_class='warn', n_jobs=None, penalty='l2', random_state=None, solver='warn', tol=0.0001, verbose=0, warm_start=False)
	Best hyperparameters applied to logistic regression with RFE.	sklearn.feature_selection.RFE<br><br>sklearn.linear_model.LogisticRegression	RFE(self, estimator, n_features_to_select=9, step=1, verbose=0)<br><br>LogisticRegression(C=0.2, class_weight=None, dual=False, fit_intercept=True,

			intercept_scaling=1, max_iter=7, multi_class='warn', n_jobs=None, penalty='l2', random_state=None, solver='warn', tol=0.0001, verbose=0, warm_start=False)
Support Vector Machine	Support Vector Classifier with Linear kernel and default parameters	sklearn.svm.SVC	SVC(self, C=1.0, kernel='linear', degree=3, gamma='auto_deprecated', coef0=0.0, shrinking=True, probability=False, tol=1e-3, cache_size=200, class_weight=None, verbose=False, max_iter=-1, decision_function_shape='ovr', random_state=None)
	Support Vector Classifier with Linear kernel and Principal component analysis (PCA) applied on input	sklearn.decomposition.PCA<br><br>sklearn.svm.SVC	PCA(copy=True, iterated_power='auto', n_components='mle', random_state=None, svd_solver='full', tol=0.0, whiten=False)<br><br>SVC(self, C=1.0, kernel='linear', degree=3, gamma='auto_deprecated', coef0=0.0, shrinking=True, probability=False, tol=1e-3, cache_size=200, class_weight=None, verbose=False, max_iter=-1, decision_function_shap

			e='ovr', random_state=None)
	Support Vector Classifier with RBF kernel and Principal component analysis (PCA) applied on input	sklearn.decomposition.PCA<br><br>sklearn.svm.SVC	PCA(copy=True, iterated_power='auto', n_components='mle', random_state=None, svd_solver='full', tol=0.0, whiten=False)<br><br>SVC(self, C=1.0, kernel='rbf', degree=3, gamma='auto_deprecated', coef0=0.0, shrinking=True, probability=False, tol=1e-3, cache_size=200, class_weight=None, verbose=False, max_iter=-1, decision_function_shape='ovr', random_state=None)
Decision Tree classifier	Decision Tree classifier with default parameters	sklearn.tree.DecisionTreeClassifier	DecisionTreeClassifier(self, criterion="gini", splitter="best", max_depth=None, min_samples_split=2, min_samples_leaf=1, min_weight_fraction_leaf=0., max_features=None, random_state=None, max_leaf_nodes=None, min_impurity_decrease=0., min_impurity_split=None, class_weight=None, presort=False)
	Hyperparameter selected decision tree	sklearn.model_selection.validation_curve<br><br>sklearn.tree.DecisionTreeClassifier	DecisionTreeClassifier(class_weight=None, criterion='gini', max_depth=3, max_features=None, max_leaf_nodes=None, min_impurity_decrease

			=0.0, min_impurity_split=1e-06, min_samples_leaf=1, min_samples_split=2, min_weight_fraction_leaf=0.0, presort=False, random_state=0, splitter='best')
	Bagged decision tree with default tree and default bagging parameters	sklearn.ensemble.BaggingClassifier<br><br>sklearn.tree.DecisionTreeClassifier	BaggingClassifier(base_estimator=DecisionTreeClassifier(class_weight=None, criterion='gini', max_depth=None, max_features=None, max_leaf_nodes=None, min_impurity_decrease=0.0,min_impurity_split=None, min_samples_leaf=1, min_samples_split=2, min_weight_fraction_leaf=0.0, presort=False, random_state=None, splitter='best'), bootstrap=True, bootstrap_features=False, max_features=1.0, max_samples=1.0, n_estimators=10, n_jobs=None, oob_score=False, random_state=None, verbose=0, warm_start=False)
	Bagged decision tree with default bagging parameters and hyperparameter selected decision tree	sklearn.ensemble.BaggingClassifier<br><br>sklearn.tree.DecisionTreeClassifier	BaggingClassifier(base_estimator=DecisionTreeClassifier(class_weight=None, criterion='gini', max_depth=3, max_features=None, max_leaf_nodes=None, min_impurity_decrease=0.0, min_impurity_split=1e-06,

			min_samples_leaf=1, min_samples_split=2, min_weight_fraction_leaf=0.0, presort=False, random_state=0, splitter='best'), bootstrap=True, bootstrap_features=False, max_features=1.0, max_samples=1.0, n_estimators=10, n_jobs=None, oob_score=False, random_state=None, verbose=0, warm_start=False)
	Bagged decision tree with hyperparameter selected bagging parameters and hyperparameter selected Decision Tree	sklearn.ensemble.BaggingClassifier<br><br>sklearn.tree.DecisionTreeClassifier	BaggingClassifier(base_estimator=DecisionTreeClassifier(class_weight=None, criterion='gini', max_depth=9, max_features='sqrt', max_leaf_nodes=None, min_impurity_decrease=0.0, min_impurity_split=None, min_samples_leaf=1, min_samples_split=2, min_weight_fraction_leaf=0.0, presort=False, random_state=None, splitter='best'), bootstrap=True, bootstrap_features=False, max_features=1.0, max_samples=1.0, n_estimators=30, n_jobs=None, oob_score=False, random_state=None, verbose=0, warm_start=False)
Random Forest Classifier	Random forest classifier with default parameters	sklearn.ensemble.RandomForestClassifier	RandomForestClassifier(bootstrap=True,

			class_weight=None, criterion='gini', max_depth=None, max_features='auto', max_leaf_nodes=None, min_impurity_decrease=0.0, min_impurity_split=None, min_samples_leaf=1, min_samples_split=2, min_weight_fraction_leaf=0.0, n_estimators='warn', n_jobs=None, oob_score=False, random_state=None, verbose=0, warm_start=False)
	Hyperparameter selected random forest classifier	sklearn.model_selection.validation_curve<br><br>sklearn.ensemble.RandomForestClassifier	RandomForestClassifier(bootstrap=True, class_weight=None, criterion='gini', max_depth=7, max_features='auto', max_leaf_nodes=None, min_impurity_decrease=0.0, min_impurity_split=None, min_samples_leaf=1, min_samples_split=2, min_weight_fraction_leaf=0.0, n_estimators=69, n_jobs=None, oob_score=False, random_state=None, verbose=0, warm_start=False)
Extra Trees classifier	Extra Trees classifier with default parameters	sklearn.ensemble import ExtraTreesClassifier	ExtraTreesClassifier(bootstrap=False, class_weight=None, criterion='gini', max_depth=None, max_features='auto', max_leaf_nodes=None,

			min_impurity_decrease =0.0, min_impurity_split=None, min_samples_leaf=1, min_samples_split=2, min_weight_fraction_leaf=0.0, n_estimators='warn', n_jobs=None, oob_score=False, random_state=None, verbose=0, warm_start=False)
	Extra trees classifier with hyperparameter selection	sklearn.model_selection .validation_curve<br><br>sklearn.ensemble import ExtraTreesClassifier	ExtraTreesClassifier(bootstrap=False, class_weight=None, criterion='gini', max_depth=25, max_features='auto', max_leaf_nodes=None, min_impurity_decrease =0.0, min_impurity_split=None, min_samples_leaf=1, min_samples_split=2, min_weight_fraction_leaf=0.0, n_estimators=80, n_jobs=None, oob_score=False, random_state=None, verbose=0, warm_start=False)
K nearest neighbors classifier	KNN classifier with default parameters	sklearn.neighbors. KNeighborsClassifier	KNeighborsClassifier(algorithm='auto', leaf_size=30, metric='minkowski', metric_params=None, n_jobs=None, n_neighbors=5, p=2, weights='uniform')
	Hyperparameter selected KNN	sklearn.neighbors. KNeighborsClassifier	KNeighborsClassifier(algorithm='auto', leaf_size=30,

			metric='minkowski', metric_params=None, n_jobs=None, n_neighbors=20, p=2, weights='uniform')
	Hyperparameter selected KNN with weights="uniform"	sklearn.neighbors. KNeighborsClassifier	KNeighborsClassifier(algorithm='auto', leaf_size=30, metric='minkowski', metric_params=None, n_jobs=None, n_neighbors=20, p=2, weights='uniform')
	Hyperparameter selected KNN with weights="distance"	sklearn.neighbors. KNeighborsClassifier	KNeighborsClassifier(algorithm='auto', leaf_size=30, metric='minkowski', metric_params=None, n_jobs=None, n_neighbors=20, p=2, weights='distance')
	Bagged hyperparameter selected KNN	sklearn.ensemble.BaggingClassifier<br><br>sklearn.neighbors. KNeighborsClassifier	BaggingClassifier(base_estimator=KNeighborsClassifier(algorithm='auto', leaf_size=30, metric='minkowski', metric_params=None, n_jobs=None, n_neighbors=20, p=2, weights='uniform'), bootstrap=True, bootstrap_features=False, max_features=1.0, max_samples=1.0, n_estimators=10, n_jobs=None, oob_score=False, random_state=None, verbose=0, warm_start=False)
	KNN with n_neighbors selected by common practice	sklearn.neighbors. KNeighborsClassifier	KNeighborsClassifier(algorithm='auto', leaf_size=30, metric='minkowski', metric_params=None, n_jobs=None, n_neighbors=237, p=2,

			weights='uniform')
MLP Classifier	MLP with default parameters	sklearn.neural_network.MLPClassifier	MLPClassifier(activation='relu', alpha=0.0001, batch_size='auto', beta_1=0.9, beta_2=0.999, early_stopping=False, epsilon=1e-08, hidden_layer_sizes=(100), learning_rate='constant', learning_rate_init=0.001, max_iter=200, momentum=0.9,n_iter_no_change=10, nesterovs_momentum=True, power_t=0.5, random_state=None, shuffle=True, solver='adam', tol=0.0001, validation_fraction=0.1, verbose=False, warm_start=False)
	MLP trained with hidden layer sizes selected using common practice	sklearn.neural_network.MLPClassifier	MLPClassifier(activation='relu', alpha=0.0001, batch_size='auto', beta_1=0.9,beta_2=0.999, early_stopping=False, epsilon=1e-08, hidden_layer_sizes=(6), learning_rate='constant', learning_rate_init=0.001, max_iter=200, momentum=0.9, n_iter_no_change=10, nesterovs_momentum=True, power_t=0.5, random_state=None, shuffle=True, solver='adam', tol=0.0001, validation_fraction=0.1, verbose=False, warm_start=False)
	Using hold out cross validation dataset to	sklearn.neural_network.MLPClassifier	MLPClassifier(activation='relu', alpha=0.0001,

	adjust the hidden layer sizes in MLP		batch_size='auto', beta_1=0.9, beta_2=0.999, early_stopping=False, epsilon=1e-08, hidden_layer_sizes=(35, 35, 35), learning_rate='constant', learning_rate_init=0.001, max_iter=1000, momentum=0.9, n_iter_no_change=10, nesterovs_momentum=True, power_t=0.5, random_state=None, shuffle=True, solver='adam', tol=0.0001, validation_fraction=0.1, verbose=False, warm_start=False)
Gradient Boosted Trees (GBT)	GBT with default parameters	sklearn.ensemble.GradientBoostingClassifier	GradientBoostingClassifier(criterion='friedman_mse', init=None, learning_rate=0.1, loss='deviance', max_depth=3, max_features=None, max_leaf_nodes=None, min_impurity_decrease=0.0, min_impurity_split=None, min_samples_leaf=1, min_samples_split=2, min_weight_fraction_leaf=0.0, n_estimators=100, n_iter_no_change=None, presort='auto', random_state=None, subsample=1.0, tol=0.0001, validation_fraction=0.1, verbose=0, warm_start=False)

	Hyperparameter selected GBT	sklearn.ensemble.GradientBoostingClassifier	GradientBoostingClassifier(criterion='friedman_mse', init=None, learning_rate=0.1, loss='deviance', max_depth=5, max_features=6, max_leaf_nodes=None, min_impurity_decrease=0.0, min_impurity_split=None, min_samples_leaf=1, min_samples_split=2, min_weight_fraction_leaf=0.0, n_estimators=50, n_iter_no_change=None, presort='auto', random_state=0, subsample=1.0, tol=0.0001, validation_fraction=0.1, verbose=0, warm_start=False)
Adaboost classifier	Adaboost classifier with default parameters	sklearn.ensemble.AdaBoostClassifier	AdaBoostClassifier(algorithm='SAMME.R', base_estimator=None, learning_rate=1.0, n_estimators=50, random_state=None)
	Adaboost with decision tree as the base classifier	sklearn.ensemble.AdaBoostClassifier	AdaBoostClassifier(algorithm='SAMME.R', base_estimator=DecisionTreeClassifier(class_weight=None, criterion='gini', max_depth=None, max_features=None, max_leaf_nodes=None, min_impurity_decrease=0.0, min_impurity_split=None, min_samples_leaf=1, min_samples_split=2,

			min_weight_fraction_leaf=0.0, presort=False, random_state=None, splitter='best'), learning_rate=1.0, n_estimators=50, random_state=None)
	Adaboost with decision tree as the base classifier<br>and hyperparameter selected boosting parameters	sklearn.ensemble.AdaBoostClassifier	AdaBoostClassifier(algorithm='SAMME.R', base_estimator=DecisionTreeClassifier(class_weight=None, criterion='gini', max_depth=3, max_features=None, max_leaf_nodes=None, min_impurity_decrease=0.0, min_impurity_split=1e-06, min_samples_leaf=1, min_samples_split=2, min_weight_fraction_leaf=0.0, presort=False, random_state=0, splitter='best'),learning_rate=1.0, n_estimators=50, random_state=None)
Voting classifier	Voting classifiers with Logistic regression and RandomForest as estimators	sklearn.ensemble.VotingClassifier<br><br>sklearn.linear_model.LogisticRegression<br><br>sklearn.ensemble.RandomForestClassifier	VotingClassifier(estimators=[('lr', LogisticRegression(C=1.0, class_weight=None, dual=False, fit_intercept=True, intercept_scaling=1, max_iter=100, multi_class='warn', n_jobs=None, penalty='l2', random_state=None, solver='warn', tol=0.0001, verbose=0, warm_start=False)), ('rf', RandomForestClassifier

			(bootstrap=True, class_weight=None, criterion='gini', max_depth=None, max_features='auto', max_leaf_nodes=None, min_impurity_decrease=0.0, min_impurity_split=None, min_samples_leaf=1, min_samples_split=2, min_weight_fraction_leaf=0.0, n_estimators='warn', n_jobs=None, oob_score=False, random_state=None, verbose=0, warm_start=False)], flatten_transform=None, n_jobs=None, voting='soft', weights=None)
	Voting classifiers with Gradient Boosting Classifier and Adaboost classifier as estimators	sklearn.ensemble.VotingClassifier<br><br>sklearn.ensemble. GradientBoostingClassifier<br><br>sklearn.ensemble.AdaBoostClassifier	VotingClassifier(estimators=[('gbt', GradientBoostingClassifier(criterion='friedman_mse', init=None, learning_rate=0.1, loss='deviance', max_depth=3, max_features=None, max_leaf_nodes=None, min_impurity_decrease=0.0, min_impurity_split=None, min_samples_leaf=1,... m='SAMME.R', base_estimator=None, learning_rate=1.0, n_estimators=50, random_state=None))], flatten_transform=None, n_jobs=None, voting='soft', weights=None)
